# How is neck dissection performed in Oral and Maxillofacial Surgery? Results of a representative nationwide survey among university and non-university hospitals in Germany

**DOI:** 10.1007/s00784-020-03622-9

**Published:** 2021-03-29

**Authors:** Andreas Pabst, Daniel G. E. Thiem, Elisabeth Goetze, Alexander K. Bartella, Michael T. Neuhaus, Jürgen Hoffmann, Alexander-N. Zeller

**Affiliations:** 1 Department of Oral and Maxillofacial Surgery, Federal Armed Forces Hospital, Rübenacherstr. 170, 56072 Koblenz, Germany; 2 Department of Oral and Maxillofacial Surgery, University Medical Center Mainz, Augustusplatz 2, 55131 Mainz, Germany; 3 Department of Oral and Maxillofacial Surgery, University Hospital Erlangen, Glückstr. 11, 91054 Erlangen, Germany; 4 Department of Oral- and Maxillofacial Surgery, University Hospital Leipzig, Liebigstr. 12, 04103 Leipzig, Germany; 5 Department of Oral and Maxillofacial Surgery, Hannover Medical School, Carl-Neuberg-Str. 1, 30625 Hannover, Germany; 6 Department of Oral and Maxillofacial Surgery, University Hospital Heidelberg, Im Neuenheimer Feld 400, 69120 Heidelberg, Germany

**Keywords:** Oncology, Oral squamous cell carcinoma, Neck dissection, Lymph node, Metastases, Survey

## Abstract

**Introduction:**

Neck dissection (ND) is a surgical procedure addressing cervical lymph nodes and metastases in patients with oral squamous cell carcinoma (OSCC). The aim of this study was to analyze clinical decisions regarding indications and variations of ND in Oral and Maxillofacial Surgery (OMFS) in Germany.

**Material and methods:**

A nationwide survey of the German Association of Oral and Maxillofacial Surgery was performed using dynamic online questionnaires including 38 questions. Data about oncological centers, case numbers, and staging procedures were collected. Relevant aspects, such as inclusion of level IIb and levels IV and V to ND, uni- vs. bilateral ND, and the influence of extra-nodal extension (ENE) of metastases on extension of ND were evaluated.

**Results:**

Eighty-four OMFS of university and non-university hospitals participated in the study (responding rate 21.4%). Sixty-six (78.57%) stated to work at certified cancer centers and 53.57% of the hospitals treated between 50 and 100 OSCC cases per year. CT and/or MRI of the head and neck was performed in most of the staging procedures. Level IIb was included by 71 (93.42%) of the participants in selective ND. Levels IV and V were included by 53 (69.74%) in node-positive neck. In solitary ipsilateral metastases (ENE−), 49 participants (62.82%) stated to perform exclusively an ipsilateral ND and 40 (51.95%) stated to perform only an ipsilateral ND in ENE+.

**Conclusion:**

This study demonstrated a high rate of certified cancer centers in Germany showing differences regarding staging procedures, indications, and extension of ND, especially in increasingly complex cases.

**Clinical relevance:**

Clinical decisions regarding ND are dependent on case-individual aspects and must be decided individually.

## Introduction

**Fig. 1:**
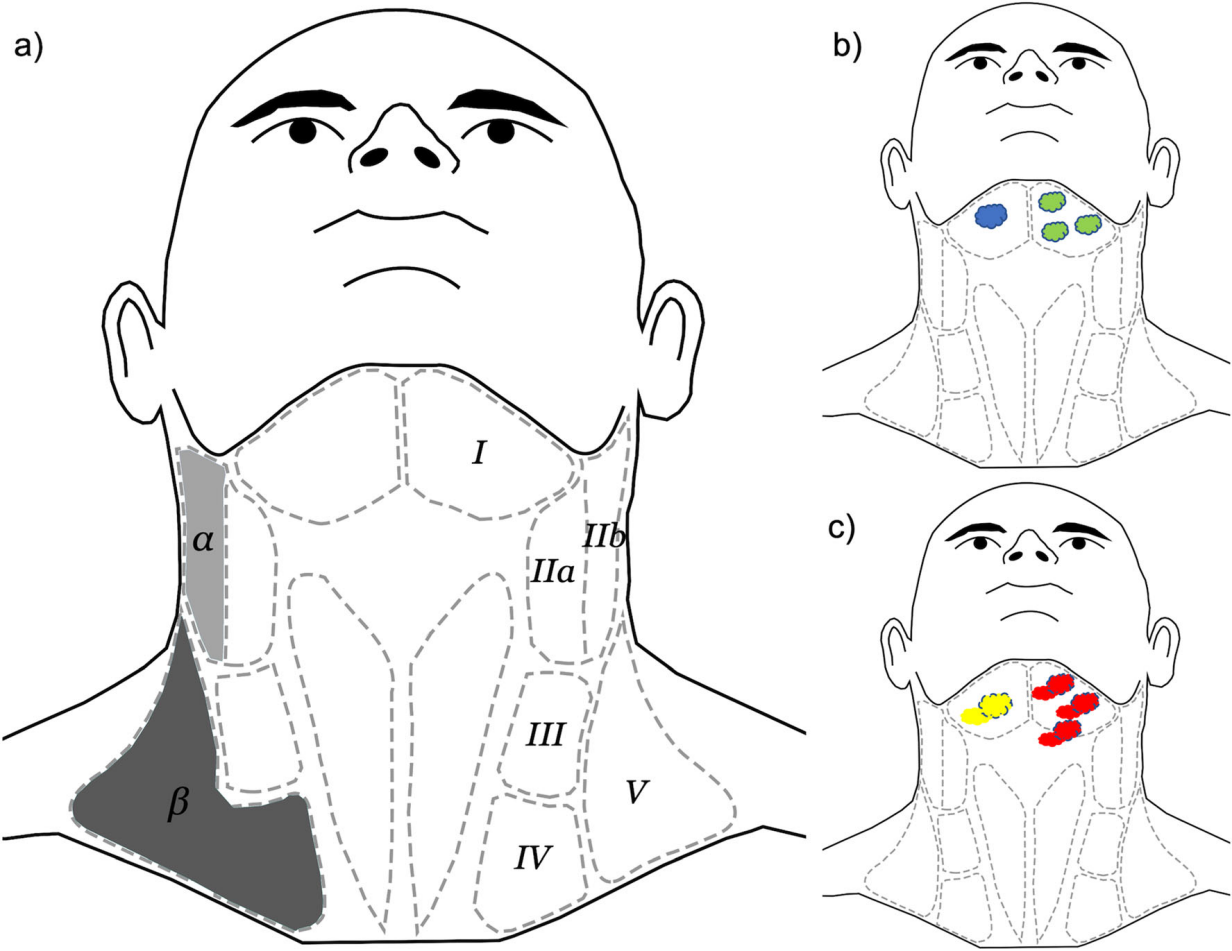
**a** Relevant nodal levels of the neck (right) and nodal levels subject to this study (left): level IIb (light gray, α) and levels IV and V (dark gray, β). **b**, **c** Display of theoretical situations within the questionnaire assuming the tumor being on the ipsilateral side. **b** Cases without ENE (−ENE) and **c** cases showing signs of ENE (ENE+). Case 1 (**b**, blue): solitary ipsilateral metastasis without ENE (−ENE), case 2 (**b**, green): multiple ipsilateral metastases without ENE (−ENE), case 3 (**c**, yellow): solitary ipsilateral metastasis with signs of ENE (ENE+), case 4 (**c**, red): multiple ipsilateral metastases with signs of ENE (ENE+)

Neck dissection (ND) is a surgical procedure addressing cervical lymph nodes and cervical lymph node metastases in the surgical treatment of oral squamous cell carcinoma (OSCC). OSCC is one of the most frequent malignancies of the head and neck with an increasing frequency and a detectable shift to women and younger patients [1, 2]. The surgical procedure of ND was originally illustrated by George W. Crile in 1906 and further developed by other surgeons made at the turn of the twentieth century [3]. According to Robbins et al., cervical lymph node locations are divided into 6 major lymph node levels (Fig. 1a), ranging from the lower edge of the mandible down to the clavicle and from the pretracheal region dorsally to the lateral throat triangle [4]. Levels Ia and Ib include the submental and the submandibular nodes; levels IIa and IIb include the upper jugular nodes; levels III and IV the middle and lower jugular nodes; level V the dorsal cervical nodes; and Level VI the pretracheal nodes [4]. Next to the location of the cervical lymph nodes according to the Robbins level, the clinical and pathological presence, localization, and frequency of positive nodes are defined by the UICC (Union International Contre le Cancer) classification (N0–N3). The last revision of the UICC classification in 2017 included stage N3b describing extra-nodal extension (ENE) of cervical lymph node metastases (ENE+), independently, from the number and location of positive nodes [5, 6]. Elective ND is the surgical procedure recommended in a diagnostic and prophylactic intention in OSCC cases clinically presenting with the N0 neck [7]. Even in clinically N0 necks, there are a relevant number of occult metastases of up to 28% that can exclusively be reliably examined by pathological evaluation [8]. There is first evidence that FDG-PET-CT (fluorodeoxyglucose-positron emission tomography-computed tomography) might be able to detect occult lymph node metastases in OSCC [9]. This is important since the number of cervical lymph node metastases is considered to be a critical predictor influencing the overall survival rate of OSCC patients [10]. Next, the decision for adjuvant radio-(chemo)therapy is also based on the presence of cervical lymph node metastases [11]. Therefore, reliable information about the cervical nodes in OSCC is required, which can be ensured by pathological examination after ND. Dealing with the clinically N0 neck in OSCC in an elective way, selective neck dissection of the supraomohyoid levels I-III is the most often conducted procedure [12]. It was reported that selective ND leads to an increase of the overall survival rate in clinically N0 neck in OSCC [13, 14]. For the node-positive neck in OSCC, there is consensus that therapeutic ND has to be performed. Dealing with the node-positive neck, a modified radical ND with preservation of non-infiltrated structures is the procedure preferred. Contrary to the selective ND, modified radical ND includes more lymph node levels and, if necessary, the removal of other cervical structures, such as the sternocleidomastoid muscle, the accessory nerve, and/or the internal jugular vein [15, 16]. The benefit of modified radical ND in advanced nodal disease (N2, N3) in OSCC is controversially discussed [17]. One reason for this controverse discussion is that ND has been reported to have a significant influence on health-related quality of life of the patients, e.g., regarding appearance and pain [18]. In this context, some authors consider sentinel lymph node (SLN) biopsy as a minimal form of ND, picking one single or a few specific, radionucleotide-labeled lymph nodes in OSCC as a substitute for ND [19]. As a major limitation, SLN biopsy entails the risk to overlook skip metastases that are associated with increased tumor size and thickness [20]. To obtain detailed preoperative information about the cervical lymph node status and the presence of positive nodes, different imaging techniques, such as computed tomography (CT) and magnetic resonance imaging (MRI), with individual advantages and limitations are used [21]. Even though ND is a frequently performed surgical procedure in OSCC in Oral and Maxillofacial Surgery (OMFS) with a significant number of national and international guidelines and literature, there is a multitude of questions that are discussed again and again. These discussions mainly focus on optimized imaging techniques for preoperative staging and the different levels included in ND in clinically node-negative necks and the node-positive neck. Other topics that have been subject to discussion are the inclusion of level IIb and levels IV and V dissection in the clinically node-negative neck and the node-positive neck, uni- vs. bilateral ND and the influence of ENE of neck metastases on ND extension [9, 22–26].

Thus, the aim of this study was to obtain information regarding indications and variations of ND and to analyze the clinical decisions behind that in OSCC in Oral and Maxillofacial Surgery departments in Germany.

## Material and methods

An online survey was conducted by the board of the German Association of Oral and Maxillofacial Surgery (DGMKG) using a dynamic online questionnaire created in SurveyMonkey (San Mateo, California, USA). Depending on the participants’ answers, additional subquestions were possible for further elaboration. Thus, participants were asked to answer 16 to 38 questions. The questionnaire was designed short and concise by not asking about generally accepted facts to keep dropout rates as low as possible. Some questions were skippable. An internal validation of the questionnaires was performed by the authors prior to extensive use. For this purpose, the questionnaire was distributed in the author departments and adjusted according to the suggestions of the test participants. A concise overview over the contents of the questionnaire is displayed in Table 1. At the end of the questionnaire, an open comment section was provided.

**Table 1:** Concise overview over the contents of the questionnaire constructed. Questions and the conditions for availability of the subquestions are given in columns. Subquestions are displayed in italic letters

Questions	Condition for subquestions
Is your department certified by the German Cancer society?	If “Yes”
How many primary OSCC cases are annually treated in your department?	If “No”
Which examinations are conducted for UICC stage I&II OSCC?	
Which examinations are conducted for UICC stage III&IV OSCC?	
Does mid-line-involvement of the tumor have therapeutically consequences regarding neck dissection?	
How is mid-line involvement defined?	
In which case do you routinely conduct a bilateral ND? (Question targeting mid-line sensitive cases)	
Do you conduct a contralateral ND for cases 1–3?	For each: If “Yes”
*If yes, does this depend on the metastasis location?*	For each: If “Yes”
*If yes, in which location?*	
Which levels are included in elective ND in your department?	
Is level IIb (Fig. 1a, α) routinely included in elective ND in your department?	If “No”
*Do you include it in elective ND for cases 1–3?*	For each: If “Yes”
*If yes, does this depend on the metastasis location?*	For each: If “Yes”
*If yes, in which location?*	
Do you include levels IV & V (Fig. 1a, β) for cases 1–4?	For each: If “Yes”
*If yes, does this depend on the metastasis location?*	For each: If “Yes”
*If yes, in which location?*	
Do you have any comments regarding this questionnaire?	

As basic data and key figures, the survey contained questions about cancer center certifications and number of OSCC cases per year. Next, diagnostic imaging of tumor staging and internal guidelines for ND procedures concerning clinical decisions regarding indications and variations of ND were collected. The consequences of these internal guidelines were evaluated by exposing the participants to several theoretical situations. To evaluate decisions about the extent of ND performed and the process of decision-making behind it, four cases based on ipsilateral OSCC, presenting single or multiple metastases without (Fig. 1b) and with (Fig. 1c) extra-nodal extension (ENE), were constructed. The cases are further clarified in detail in the corresponding figure legend (Fig. 1).

In total, 393 department heads and senior consultants of university and non-university hospitals of Oral and Maxillofacial Surgery were contacted via email by the board and head office of the DGMKG (Hofheim, Germany). Participants were invited to take part in this survey on an anonymous basis. To ensure anonymity, questionnaires did not contain possibly identifying questions and responses were not evaluated by each specific workplace. The surveys were open between June 29 and August 14, 2020. Two reminders were sent to the participants 1 and 2 weeks after starting the survey. Results were collected using SurveyMonkey, analyzed using Wizard for Mac 1.9.42 by Evan Miller. Independence of nominal variables was evaluated using chi-squared and *Z*-test. *p* values < 0.05 were considered significant and highlighted with an asterisk (*). Results were illustrated in Numbers (Apple, Cupertino, California, USA), Excel, and Powerpoint for Mac (Microsoft, Redmont, Washington, USA).

Due to conditional questions of this dynamic questionnaire, not all participants answered all 38 questions. Thus, the number of participants stating each fact is displayed as an integral number, and results are additionally displayed in parentheses as the percentage of all participants answering the specific question.

## Results

### General data

**Fig. 2:**
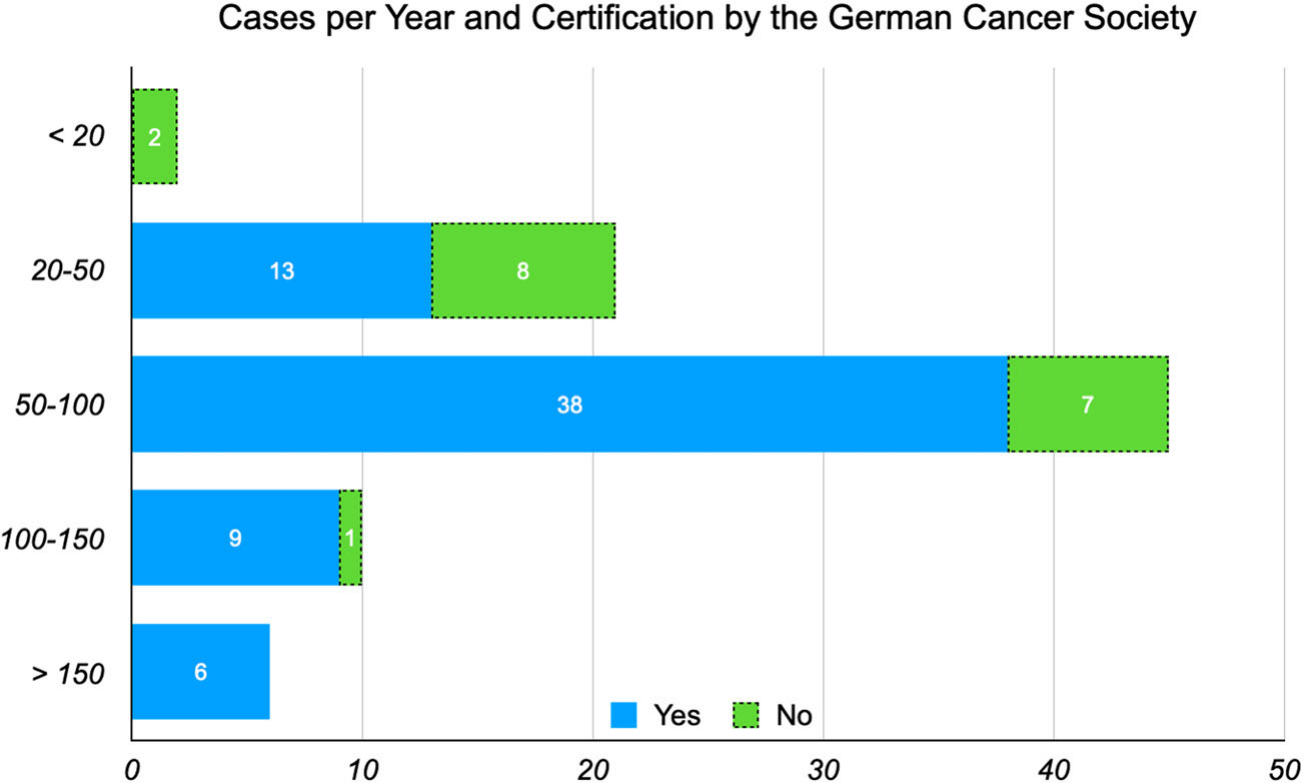
Numbers of oral squamous cell carcinomas (OSCC) treated per year (*y*-axis) and number of participants stating that range. Participants working at a hospital certified by the German Cancer Society are displayed in blue, others in green (dotted outline). Treated cases per year were significantly different depending on whether a hospital was certified by the German Cancer Society or not (chi-square, *p* = 0.007)

Of the 393 department heads and senior consultants, 84 returned questionnaires. All 84 were sufficiently filled in to be included. The responding rate was 21.4%. Sixty-six (*n* = 66/84, 78.57%) stated that they worked at a hospital certified by the German Cancer Society (Deutsche Krebsgesellschaft, DKG). Eighteen (*n* = 18/84, 21.43%) stated that their place of work was currently not officially certified by the German Cancer Society. In most cases (*n* = 45/84, 53.57%), the hospital participants treated between 50 and 100 OSCC per year. 2.38% (*n* = 2/84) treated up to 20 cases, 25.00% (*n* = 21/84) treated 20–50 cases, and 7.14% (*n* = 6/84) treated over 150 cases per year. Whether a hospital was certified by the German Cancer Society or not significantly correlated with the number of cases treated per year (chi-square, *p* = 0.007). The numbers and certification by the German Cancer Society are displayed in Fig. 2.

### Staging

**Fig. 3:**
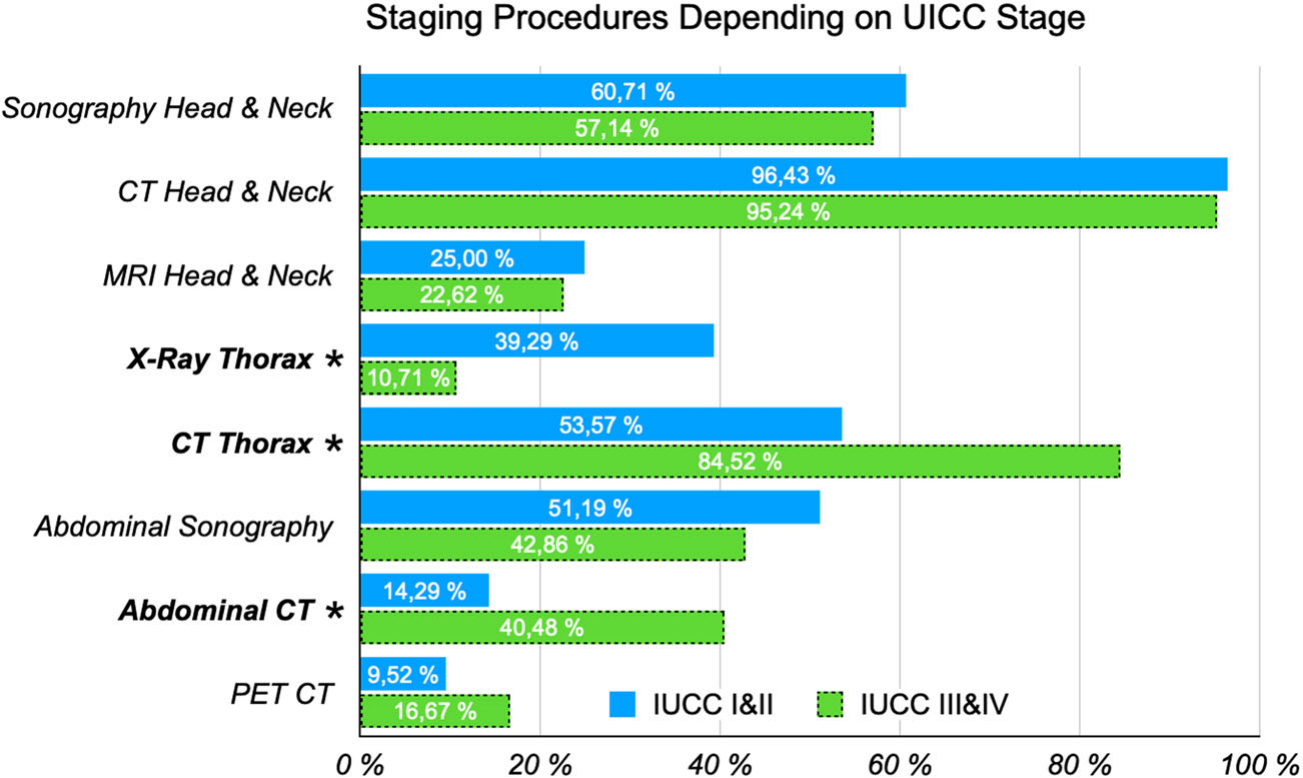
Changes in preoperative staging procedures dependent on the UICC stage. Procedures to be conducted are displayed in the *y*-axis. The percentage of participants stating they would conduct these procedures for UICC stages I and II are displayed in the top bar (blue), for UICC stages III and IV in the lower bar (green, dotted outline). Statistically significant differences depending on the categories are marked with an asterisk (*)

For evaluation of preoperative staging protocols and procedures, it was discriminated between procedures conducted for UICC stages I and II and those for UICC stages III and IV. All (*n* = 84) provided information about their staging procedures and all stated that they would routinely carry out an examination for detection of cervical metastases for all stages. For UICC stages I and II, 81 participants (96.43%) stated they would conduct a computer tomography (CT) scan of the head and neck, 51 performed sonography of the neck (60.71%), 46 carried out a CT scan of the thorax (54.76%), 43 conducted abdominal sonography (51.19%), 32 carried out a thorax X-ray (38.10%), 21 a magnetic resonance imaging (MRI) scan of the head and neck (25.00%), 12 an abdominal CT (14.29%), and 8 a PET-CT scan (9.52%). For UICC stages III and IV, 80 participants (95.24%) stated they would carry out a CT scan of the head and neck, 48 performed sonography of the neck (57.14%), 71 conducted a CT thorax (84.52%), 36 performed abdominal sonography (42.86%), 9 conducted a thorax X-ray (10.71%), 19 performed a MRI scan of the head and neck (22.62%), 34 an abdominal CT (40.48%), and 14 carried out a PET-CT scan (16.67%). CT scans of the thorax and abdomen (*Z*-test, each *p* < 0.001) were conducted significantly more often in UICC stage III and IV cancer than in UICC stage I and II cancer. Conventional thorax X-rays were carried out significantly less often (*p* < 0.001). Changes in preoperative staging procedures dependent on the UICC stage are highlighted in Fig. 3.

### Indications for neck dissection

Sixty-five participants stated (*n* = 65/78, 83.33%) they would conduct a bilateral neck dissection in case of a midline involvement of the tumor. Thirteen stated they would not routinely do this (*n* = 13/78, 16.67%). Prior to being asked about decisions for strictly circumscribed cases as displayed in Fig. 1b, c, participants were asked to provide information about the definition of midline involvement used within their departments. Several different situations were possible to be considered as midline involvement. This was stated to meet the definition if the OSCC clinically exceed the midline (*n* = 61/78, 78.21%), OSCC radiologically exceeded the midline (*n* = 57/78, 73.08%), OSCC touched the midline clinically without exceeding it (*n* = 42/78, 53.58%), or OSCC touched the midline radiologically without exceeding it (*n* = 40/78, 51.28%). Twenty-four participants (*n* = 24/78, 30.77%) indicated a midline involvement if the OSCC was radiologically 5 mm apart from the midline; six (*n* = 6/78, 7.69%) stated this was also true for distances of 10 mm. Participants were asked to provide information about when they would perform bilateral ND with respect to midline involvement. Eight participants (*n* = 8/78, 10.26%) stated a bilateral ND would be performed regardless of the tumor location and extension. Four (*n* = 4/78, 5.13%) stated they would perform a bilateral ND for cases without crossing lymphatic drainage pathways (e.g., the cheeks) if the midline was not involved, 39 (*n* = 39/78, 50.00%) stated they would do so if the midline was involved. Fifty-three participants (*n* = 53/78, 67.95%) stated they would perform a bilateral ND for cases with crossing lymphatic drainage pathways (e.g., tongue) if the midline was not involved; 65 (*n* = 65/78, 83.33%) stated they would do so if the midline was involved.

### Extension and modifications of neck dissection

Elective ND was stated to include level I by 74 participants (*n* = 74/76, 98.68%); levels II and III were included by each 75 participants (*n* = 75/76, 98.68%). Level IV was included by 15 (*n* = 15/76, 19.74%) and level V by 3 (*n* = 3/76, 3.95%). Level IIb was included by 71 (*n* = 71/76, 93.42%) of the participants. Five participants (*n* = 5/76, 6.58%) did not include level IIb by default. All further questions regarding extent and modifications of neck dissection were designed utilizing the predescribed four different theoretical cases (Fig. 1b, c).

### First case (blue)

**Fig. 4:**
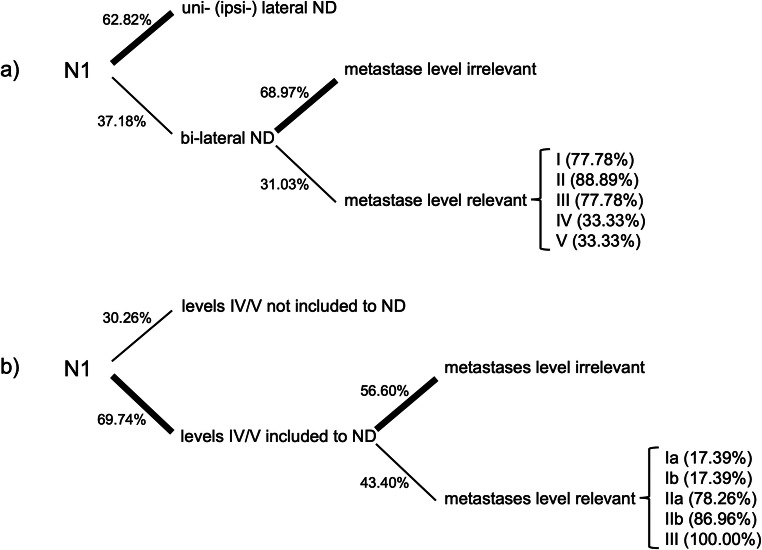
Decision tree concerning the clinical decisions for **a** uni- vs. bilateral ND and **b** inclusions of levels IV and V to ND in solitary ipsilateral metastasis ENE− (N1)

For the first case, simulating a solitary ipsilateral metastasis without ENE (Fig. 1b, blue), 49 participants (*n* = 49/78, 62.82%) stated they would only perform an ipsilateral ND. Twenty-nine participants (*n* = 29/78, 37.18%) stated they would consider a bilateral ND. Of these, 20 (*n* = 20/29, 68.97%) stated location of the metastasis would not influence their decision; 9 (*n* = 9/29, 31.03%) stated that the metastasis location would influence their decision as follows: bilateral ND would be performed if metastases were located in levels I (*n* = 7/9, 77.78%), II (*n* = 8/9, 88.89%), III (*n* = 7/9, 77.78%), IV (*n* = 3/9, 33.33%), and V (*n* = 3/9, 33.33%). Regarding the inclusion of level IIb, the 5 participants that stated they would not include it by default were asked to provide more detailed information. All 5 (100%) stated they would include it in case of a single ipsilateral metastasis without ENE (Fig. 1b, blue) if this was located in level II, III, or V. Each 3 of 5 participants (60%) stated they would include level IIb if the metastasis was located in level I or IV. For the first case, levels IV and V were not included by 23 (23/76, 30.26%). Fifty-three (53/76, 69.74%) did however include levels IV and V. Of these participants, 30 (*n* = 30/53, 56.60%) stated the metastasis location would not influence their decision; 23 (*n* = 23/53, 43.40%) stated that the metastasis location would influence their decision. In these cases, they would include levels IV and V if the metastasis was located in level Ia or Ib (each *n* = 4/23, 17.39%), IIa (*n* = 18/23, 78.26%), IIb (*n* = 20/23, 86.96%), and III (*n* = 23, 100.00%). Figure 4 is illustrating the relevant decisions made in case 1.

### Second case (green)

**Fig. 5:**
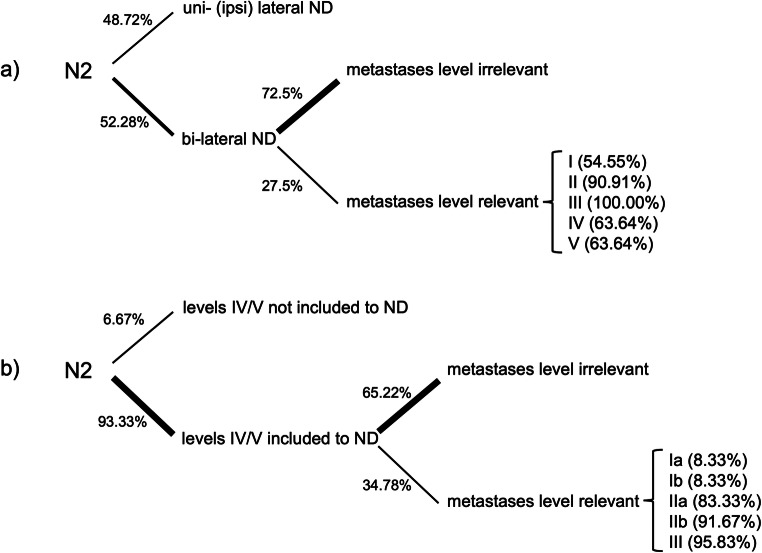
Decision tree concerning the clinical decisions for **a** uni- vs. bilateral ND and **b** inclusions of levels IV and V to ND in multiple ipsilateral metastases ENE− (N2)

For the second case, simulating multiple ipsilateral metastases without ENE (Fig. 1b, green), 38 participants (*n* = 38/78, 48.72%) stated they would only perform an ipsilateral ND. Forty participants (*n* = 40/78, 52.28%) stated they would consider a bilateral ND. Of these, 29 (*n* = 29/40, 72.50%) stated location of the metastasis would not influence their decision; 11 (*n* = 11/40, 27.50%) stated that the metastasis location would influence their decision as follows: bilateral ND would be performed if metastases were located in levels I (*n* = 6/11, 54.55%), II (*n* = 10/11, 90.91%), III (*n* = 11/11, 100.00%), IV (*n* = 7/11, 63.64%), and V (*n* = 7/11, 63.64%). It was additionally asked, which location of metastases in neck levels would not result in bilateral ND. This was stated for levels I (*n* = 5/11, 45.45%), II (*n* = 1/11, 9.09%), III (*n* = 0/11, 00.00%), IV (*n* = 3/11, 27.27%), and V (*n* = 3/11, 27.27%). Three participants (*n* = 3/11, 27.27%) stated that this was not true for any of the levels. Five participants, who initially stated to not include level IIb by default, answered they would include IIb in case of multiple ipsilateral metastases without ENE (Fig. 1b, green) if they were located in levels II, III, or V (*n* = 5/5, 100%). Also, in this group, 4 participants (*n* = 4/5, 80%) stated they would include level IIb if the metastasis was located in levels I or IV. For the second case, levels IV and V were not included by 5 (*n* = 5/75, 6.67%). Seventy (*n* = 70/75, 93.33%) did however include levels IV and V. Of these participants, 45 (*n* = 45/69, 65.22%) stated the metastasis location would not influence their decision, 24 (*n* = 24/69, 34.78%) stated that the metastasis location would influence their decision. In these cases, they would include levels IV and V, if the metastases were located in levels Ia or Ib (each *n* = 2/24, 8.33%), IIa (*n* = 20/24, 83.33%), IIb (*n* = 22/24, 91.67%), and III (*n* = 23/24, 95.83%). Figure 5 is illustrating the relevant decisions made in case 2.

### Third case (yellow)

**Fig. 6:**
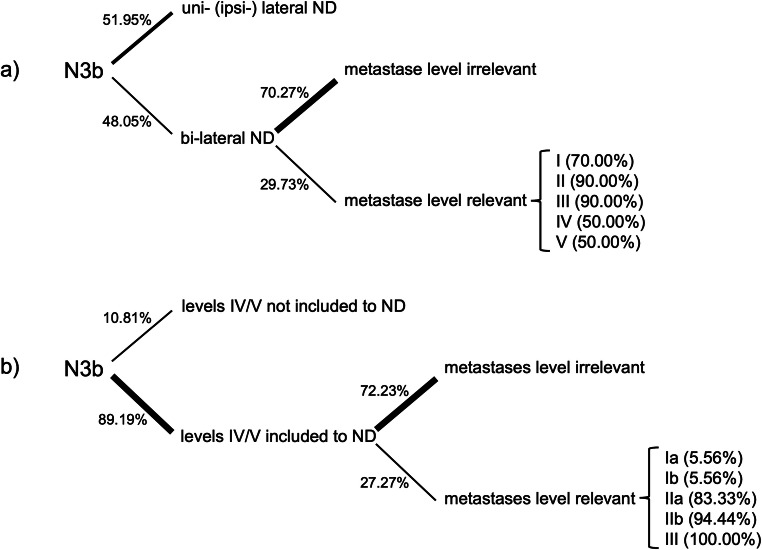
Decision tree concerning the clinical decisions for **a** uni- vs. bilateral ND and **b** inclusions of levels IV and V to ND in solitary ipsilateral metastasis ENE+ (N3b)

For the third case, simulating a solitary ipsilateral metastasis with signs of ENE (Fig. 1c, yellow), 40 participants (*n* = 40/77, 51.95%) stated they would only perform an ipsilateral ND. Thirty-seven participants (*n* = 37/77, 48.05%) stated they would consider a bilateral ND. Of these, 26 (*n* = 26/37, 70.27%) stated location of the metastasis would not influence their decision; 11 (*n* = 11/37, 29.73%) stated that the metastasis location would influence their decision. This was further specified by ten participants as follows: bilateral ND would be performed if the metastasis was located in levels I (*n* = 7/10, 70.00%), II (*n* = 9/10, 90.00%), III (*n* = 9/10, 90.00%), IV (*n* = 5/10, 50.00%), and V (*n* = 5/10, 50.00%). Regarding the inclusion of level IIb, all 5 participants that stated they would not include it by default stated they would include it in case of a single ipsilateral metastasis with ENE (Fig. 1c, yellow), if this was located in level I, II, III, or IV. Four of these participants (*n* = 4/5, 80%) stated they would include level IIb, if the metastasis was located in level V. For the third case, levels IV and V were not included by 8 (*n* = 8/74, 10.81%). Sixty-six (*n* = 66/74, 89.19%) did however include levels IV and V. Of these participants, 48 (*n* = 48/66, 72.23%) stated the metastasis location would not influence their decision. 18 (*n* = 18/66, 27.27%) stated that the metastasis location would influence their decision. In these cases, they would include levels IV and V, if the metastasis was located in level Ia or Ib (each *n* = 1/18, 5.56%), IIa (*n* = 15/18, 83.33%), IIb (*n* = 17/18, 94.44%), or III (*n* = 18/18, 100.00%). Figure 6 is illustrating the relevant decisions made in case 3.

### Fourth case (red)

**Fig. 7:**
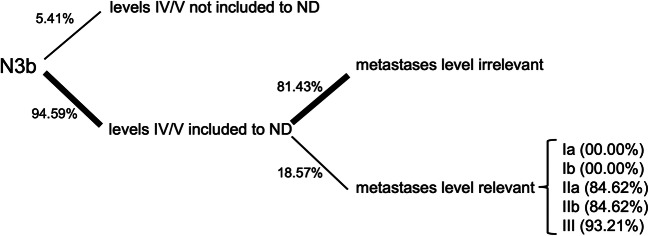
Decision tree concerning the clinical decisions for inclusions of levels IV and V to ND in multiple ipsilateral metastases ENE+ (N3b)

For the fourth case, simulating multiple ipsilateral metastases with signs of ENE (Fig. 1c, red), only questions about inclusion of levels IV and V were asked, as it was assumed to be generally accepted that a bilateral ND should be conducted and level IIb should be included. Levels IV and V were not included by 4 (*n* = 4/74, 5.41%). Seventy (*n* = 70/74, 94.59%) did however include levels IV and V. Of these participants, 57 (*n* = 57/70, 81.43%) stated the metastasis location would not influence their decision; 13 (*n* = 13/74, 18.57%) stated that the metastasis location would influence their decision. In these cases, they would include levels IV and V, if the metastases were located in level Ia or Ib (each *n* = 0/13, 0.00%), IIa or IIb (each *n* = 11/13, 84.62%), or level III (*n* = 12/13, 93.21%). Figure 7 is illustrating the relevant decisions made in case 4.

### Comparison of level inclusion and decision-making

**Fig. 8:**
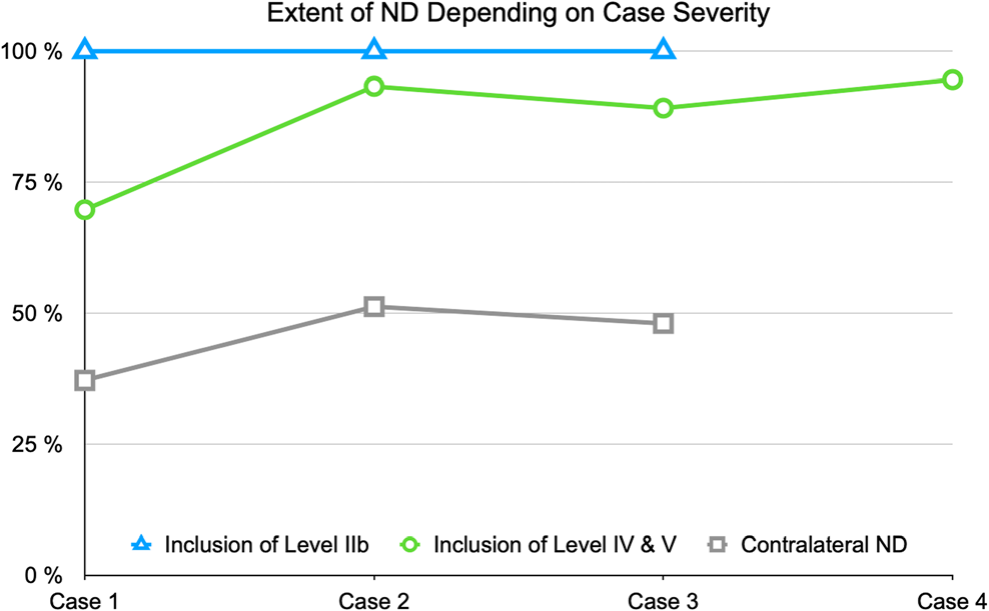
Extent of ND depending on case severity. Rates of inclusion in percent of level IIb (blue, triangles) and levels IV and V (green, circles) and indication for contralateral ND (gray, squares) are given in the *y*-axis. A trend for rising inclusion of levels IV and V and indications for contralateral ND was seen

**Fig. 9:**
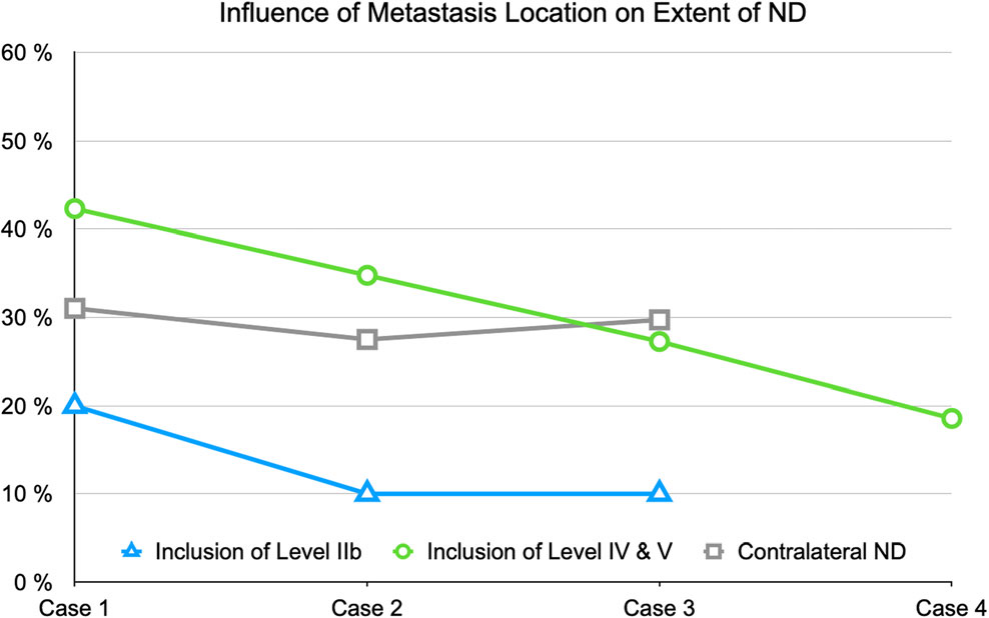
Influence of metastasis location on the extent of ND: rates of participants stating that metastasis location would influence their decision for inclusion of level IIb (blue, triangles) and levels IV and V (green, circles) and indication for contralateral ND (gray, squares) are given in the *y*-axis. Metastasis location became less important with increasing case severity especially for levels IV and V

The results regarding inclusion of levels IIb and IV/V and contralateral ND for cases 1–4 were compared with respect to the increasing case severity from case 1 to case 4. The extent of ND depending on case severity and the association of metastasis location on the extent of ND are graphically displayed in Figs. 4 and 5. A trend for rising inclusion of levels IV/V and indications for contralateral ND was seen (Fig. 8). Metastasis location became less important with increasing case severity especially for levels IV and V (Fig. 9).

## Discussion

This study addresses clinical decision-making regarding indications and variations of ND in Oral and Maxillofacial Surgery (OMFS) in Germany on the base of representative information obtained from 84 department heads and senior consultants. ND is the surgical procedure preferred to deal with the node-negative and node-positive neck in OSCC [13, 27]. High-quality (inter-)national guidelines for the diagnosis, treatment, and aftercare of OSCC and head and neck cancer and even the lymph node management are available [28–30]. The German guideline for OSCC treatment increased the quality of therapy and the number of certified cancer centers in Germany [30]. About 80% of the survey participants stated to work at a certified cancer center, illustrating the overall high quality of OSCC treatment in Germany. Unfortunately, guidelines are not able to define cancer treatment—such as the neck management—down to the last detail. This might be caused by a lack of evidence for some aspects, such as the inclusion of level IIb to selective ND in clinically N0 neck, that is, controversially discussed [25, 31, 32]. Even some definitions that are relevant for the treatment of OSCC are defined in such a vague manner. Up to date, there is no generally valid definition regarding midline exceeding of OSCC, such as often seen in OSCC of the floor of the mouth or the tongue. In this survey, most of the participants (> 73%) stated that midline exceeding is defined when the tumor exceeds the midline clinically or radiologically, followed by touching the midline clinically or radiologically (> 51%). Therefore, clinical or radiological involvement seems to be a leading reason to define midline involvement. This led to bilateral ND in 83% of the cases. Yet, with respect to the tumor microstructure, the midline maybe defined more critically. Kudo et al. visualized the tumor invasion front (TIF) in tongue cancer three-dimensionally and demonstrated that the TIF contained spiculae extending into the surrounding tissues [33]. Even every surgical OSCC resection should contain a sufficient safety margin. It has to be critically discussed how OSCC cases with clinically or radiologically close contact to the midline without touching or exceeding them should be classified regarding the “midline status.”

At the time of the initial OSCC diagnosis, about 38% of the patients already show cervical lymph node metastases [34]. Therefore, detailed information about the clinical cervical lymph node status and the possible presence of positive nodes is indispensable to plan the further treatment. In this context, a multitude of different imaging techniques is used to visualize cervical lymph nodes, such as ultrasound, CT, and MRI as well as FDG-PET-CT and PET MRI-scans [35]. Overall, ultrasound scan is widely recognized as a reliable technique to visualize cervical lymph nodes and to detect positive nodes in OSCC [36, 37]. Native ultrasound scan might be combined with the application of intravenous contrast agents and elastography to further improve cervical lymph node assessment [38]. CT and MRI scans are roughly comparable concerning cervical lymph node imaging and −metastases detection, even in the detection of ENE+ metastases [39]. Rasse reported CT and MRI scan sensitivities of 82% and 80% and CT and MRI specifities of 85% and 79% concerning the detection of positive nodes, respectively [40]. PET-CT scan is controversially discussed. There is evidence that PET-CT scan is currently not superior to CT/MRI scan in the clinically node-negative neck [41]. On the other hand, comparing CT/MRI vs. PET-CT scans, it was demonstrated that FDG-PET-CT is superior to CT/MRI imaging to detect occult neck metastases [9]. Heusch et al. further illustrated that FDG-PET MRI scan combined with diffusion-weighted imaging (DWI) is not superior to ultrasound and FDG-PET-CT scans in the detection of cervical lymph node metastases [42, 43]. In this survey, the most frequently used imaging technique for staging the head and neck in OSCC was CT, followed by ultrasound and MRI scans. The high rate of CT scans might be due to the speedy access, the widespread availability, and the short scanning time. On the contrary, MRI scan is more expensive, and the scanning time is significantly longer compared with CT scan that is not tolerated by all patients. Interestingly, ultrasound scans were only performed by about 60% of the survey participants in OSCC UICC stage I/II and stage III/IV cases. Even ultrasound scan is widely available, inexpensive, and free of radiation exposure to the patient, its relevant limitations are the poor comparability with previous ultrasound examinations and its dependence on the investigator and their experience regarding this technique. Otherwise, if documentation of the relevant structures is done as in GCP (Good Clinical Practice) of ultrasound recommended, examinations might also be reproducible. Ultrasound examination might help the surgeon to get an additional impression of the situs and the individual neck anatomy independently of the imaging techniques (e.g., CT, MRI, and PET-CT) further performed. It should be kept in mind that even CT, MRI, and PET-CT examinations can be dependent from the radiologist. FDG-PET-CT scan is becoming increasingly important, such as in the detection of occult metastases, radiotherapy planning, and treatment response [44, 45]. PET-CT scan is time-consuming, expensive, and has a limited availability. With respect to the whole-body staging, significant changes were seen concerning the thorax and abdominal staging. Concerning thorax staging, there were significant changes comparing OSCC UICC stages I/II vs. III/IV with a significantly increased frequency of CT scans. This might be due to the higher risk of pulmonary metastases or secondary tumors in advanced OSCC UICC stages and is in accordance with the German guidelines.

Extra-nodal extension (ENE) of cervical lymph node metastases has been figured out to be an important prognostic factor in patients’ overall survival (OS) [46, 47]. Next, it is a relevant risk factor for a recurrent disease, metachronous lymph node metastases as well as distant metastases [10, 46, 48]. According to García et al., ENE+ nodes are detectable in 50.1% of ND specimens. Introducing N3b in the 8th edition of the TNM classification of the head and neck tumors in 2017 caused a significant upstaging from N2 to N3b of about 58.4% in OSCC cases [5, 49]. In fact, it represents a major prognostic factor, worsening the pathological N-classification in lymph nodes with a diameter < 3 cm from N1 (ENE−) to N2a (ENE+) and in lymph nodes with a diameter > 3 cm from N2a (ENE−) to N3b (ENE+). It ultimately leads to a consecutive increase of the UICC stadium III to IVA and UICC stadium IVA to IVB, respectively [5]. However, to authors’ best knowledge, there is no solid evidence in literature describing ENE without concurrent risk factors to be the reason for contralateral lymph node metastasis. There are several further parameters, such as tumor location, grading, number, and size of lymph node metastases as well as depth of invasion [46, 50]. Those factors should be also considered when considering extension of neck dissection to the contralateral side without clinical signs of metastases. Next, there is no reliable data whether ND extension or an increased surgical radicality (such as radical ND) in N3b OSCC is able to improve OS. Liao et al. reported that pN+ (≥ 8 positive nodes, ≥ 5 ENE+) nodes, metastases localized in levels IV and V, and depth of invasion (DOI) are adverse prognostic factors for OS. Next, this study illustrated that pN+ subgroups (≤ 7 positive nodes, ≤ 4 ENE+) showed better outcome than higher burden pN+ (≥ 8 positive nodes, ≥ 5 ENE+) [26]. Also, the negative influence of positive nodes in OSCC on OS is proven, ENE+ might not influence the OS [51]. It might be possible that the total number of positive nodes and maybe even the location is more relevant than ENE+ for OS. Lymph node metastases in levels IV and V are rare [52, 53]. Otherwise, these locations can be associated with a decreased OS in cases of positive nodes [54]. Whereas the adjustment of adjuvant therapy to ENE of cervical metastases is suggested in the guidelines, to be escalated from conventional radiotherapy to radio-chemotherapy, the surgical consequences remain vague [55]. In the participating cohort to this study, we noticed that the presence of an ENE+ would lead in the majority of cases (62%) only to ipsilateral ND. However, most surgeons would dissect also levels IV and V. The appearance of multiple positive lymph nodes was rated with almost the same clinical consequences as the appearance of extra-nodal growth. In patients with multiple cervical metastases, 52% of the surgeons stated that they would escalate the ND to bilateral ND. Likewise, in patients with extra-nodal growth, 48% of the surgeons would dissect both sites of the neck. Levels IV and V were also removed in the majority of cases in the two described scenarios. The fact that only half of the surgeons consider bilateral ND in patients with ENE+ and/or multiple cervical metastases shows the dilemma that the benefit of a surgical escalation of therapy needs to be weighted out with potential postoperative comorbidity following a bilateral neck dissection.

Extending therapeutic ND to level IIb and levels IV and V is associated with an additional risk of intra- and postoperative complications. Besides general risks common for all ND procedures, dissection of the submuscular recess (level IIb nodes) involves the risk of permanent damage to the spinal accessory nerve and functional impairment due to manipulation [56, 57]. As damage of the spinal accessory nerve is known to be associated with increased shoulder and neck pain and the afore-described functional impairment, it seemed surprising that most survey participants stated they would routinely include level IIb [58]. However, salvage ND due to the occurrence of later metastasis and initial renunciation of elective ND revealed a clear deterioration of the prognosis [13]. From this point, the necessity of level IIb clearance has to be critically discussed depending on the respective tumor localization and extent and the clinical cervical lymph node status. Depending on the location of the primary tumor in the oral cavity, level IIb is rarely affected in the case of a clinically inconspicuous neck (in up to 5% of cases) and is almost without exception restricted to OSCC of the tongue [59–61]. In this context, Garreau et al. found no lymph node metastases in a collective of 138 OSCC and 199 ND, regardless of tumor size (40.6% pT2) and location (34% OSCC of the tongue) [62]. For tongue cancers, therefore, level IIb clearance can be considered to be required, while for oral cancers, level IIb clearance may be considered omitted if there is no other evidence of lymph node metastases [31]. OSCC of the tongue metastasize primarily to lymph node levels I and II, with only a small proportion (6–12%) showing contralateral or bilateral lymph node metastases depending on the tumors’ proximity to the midline and its aggressiveness [63, 64]. The far dorsal location of tongue carcinomas seems to be related to the increased incidence of ipsilateral level IIb involvement and contralateral metastatic spread due to a more pronounced lymphatic branching in this area [65–67]. Besides the inclusion of level IIb, participants were asked in detail about the inclusion of levels IV and V, as well. Similar to level IIb, sparing of level V has been shown to be associated with less shoulder or neck pain and fewer physical problems [58]. Furthermore, dissection of levels IV and V comes with a variety of possibilities of complications such as chyle fistulas due to rupture of the thoracic duct [68]. As with regard to the need to include levels IV and V in a clinically node-positive situation, the current evidence is not sufficient to assess the importance of clearance in terms of prognosis and overall survival. This in turn is based on the fact that in cases of lymph node metastases of levels I–III, the probability for positive lymph nodes in level IV is given at 7–17% and for level V at 0–6% [69]. The high rates of inclusion of levels IV and V thus seem to be questionable.

## Conclusions

This study demonstrated a relevant number of OMFS departments with an overall high caseload certified by the German Cancer Society in Germany to ensure nationwide patient care at the highest medical level. Even there were visible differences concerning the preoperative imaging techniques and procedures in the context of tumor staging, the preferred imaging techniques fall within (inter-) national guidelines and are overall suitable and accepted to visualize cervical lymph nodes (−metastases). Prospectively, it might be of high interest and clinical relevance, whether existing and innovative imaging techniques might be able to detect lymph node metastases and especially occult lymph node metastases more precisely. The survey even highlighted differences regarding indications and extension of ND in node-negative and node-positive OSCC, especially in increasingly complex cases. Even patient-specific aspects, such as comorbidities, were excluded in the questionnaire, clinical decisions regarding indications, extension, and procedures of ND in OSCC cases and are strictly dependent from a multitude of patient-individual aspects and must be decided individually in each case.

To conclude, this survey demonstrated that there is an ongoing need for nationwide prospective clinical trials with a focus on the indications and the extension of ND in OSCC in line of the ideal treatment of our patients. Future challenges might be ND extension and non-surgical treatments of the node-positive neck [17, 27]. The high OSCC case numbers and the high ND frequency in Germany might even facilitate nationwide retrospective study designs.
