# Correction to: How is neck dissection performed in Oral and maxillofacial surgery? Results of a representative nationwide survey among university and non-university hospitals in Germany

**DOI:** 10.1007/s00784-021-03963-z

**Published:** 2021-05-14

**Authors:** Andreas Pabst, Daniel G. E. Thiem, Elisabeth Goetze, Alexander K. Bartella, Michael T. Neuhaus, Jürgen Hoffmann, Alexander-N. Zeller

**Affiliations:** 1 Department of Oral and Maxillofacial Surgery, Federal Armed Forces Hospital, Rübenacherstr. 170, 56072 Koblenz, Germany; 2 Department of Oral and Maxillofacial Surgery, University Medical Center Mainz, Augustusplatz 2, 55131 Mainz, Germany; 3 Department of Oral and Maxillofacial Surgery, University Hospital Erlangen, Glückstr. 11, 91054 Erlangen, Germany; 4 Department of Oral- and Maxillofacial Surgery, University Hospital Leipzig, Liebigstr. 12, 04103 Leipzig, Germany; 5 Department of Oral and Maxillofacial Surgery, Hannover Medical School, Carl-Neuberg-Str. 1, 30625 Hannover, Germany; 6 Department of Oral and Maxillofacial Surgery, University Hospital Heidelberg, Im Neuenheimer Feld 400, 69120 Heidelberg, Germany

**Correction to: Clinical Oral Investigations.**

10.1007/s00784-020-03622-9

During a routine check, the Open Access coordinator noticed that article 10.1007/s00784-020-03622-9 was published without OA, although the author choose Open Access. Additionally, the article was approved under DEAL on 08/10/2020.

The original article has been corrected.

